# New Insights: P.I.G in Preterm Infants With Isolated PDA and Severe Pulmonary Hypertension

**DOI:** 10.1155/crpe/6268296

**Published:** 2025-11-30

**Authors:** Nadya Ben Fadel, Elham Almoli, Joseph de Nanassy, Sally Mashally

**Affiliations:** ^1^Department of Pediatrics, Division of Neonatology, Faculty of Medicine, Children's Hospital of Eastern Ontario, University of Ottawa, Ottawa, Ontario, Canada; ^2^Faculty of Medicine, Children's Hospital of Eastern Ontario Research Institute, University of Ottawa, Ottawa, Ontario, Canada; ^3^Department of Pathology and Laboratory Medicine, Faculty of Medicine, Children's Hospital of Eastern Ontario, University of Ottawa, Ottawa, Ontario, Canada

**Keywords:** patent ductus arteriosus (PDA), preterm, pulmonary hypertension (PHT), pulmonary interstitial glycogenolysis (P.I.G)

## Abstract

We present a case of a premature infant who had a persistent patent ductus arteriosus (PDA) and subsequently developed severe pulmonary hypertension (PHT) and respiratory failure. A lung biopsy was performed during PDA ligation, revealing a consistent thickening of the interstitial tissue. The biopsy also showed the presence of immature interstitial cells containing high amounts of cytoplasmic glycogen, indicative of pulmonary interstitial glycogenosis (P.I.G). There was no conclusive evidence of maturational arrest, infection, alveolar proteinosis, or alveolar capillary dysplasia. While the association between P.I.G and PHT has been documented in case reports involving children with congenital heart disease (CHD), the majority of these cases involved full-term infants and older children. Notably, there have been no reports on the diagnosis of P.I.G in infants with isolated PDA, without any other congenital heart conditions. This expands the existing knowledge of P.I.G, highlighting the diagnostic value of lung biopsy in premature infants with long-standing PDA and severe PHT exhibiting similar characteristics.

## 1. Case Report

 A premature female infant weighing 920 g was born via emergency Caesarean section at 27 weeks gestation due to significant bleeding from placental abruption. The infant was born vigorous with APGAR scores of 6, 8, and 8 at 1, 5, and 10 min, respectively. Initially, the infant received positive pressure ventilation (PPV) for 1 min, followed by continuous positive airway pressure (CPAP) of 7 mmHg. The highest oxygen concentration required was 60%. In accordance with NICU policy, the baby received a 10-day course of prophylactic hydrocortisone for bronchopulmonary dysplasia (BPD) prevention during the first week of life.

At 4 days of age, the baby underwent initial screening echocardiography, revealing normal size and function of both ventricles and a moderate restrictive patent ductus arteriosus (PDA) measuring 1.6-mm shunting left-to-right. Subsequent echocardiography at 1 week of age showed similar results and no signs of early pulmonary hypertension (PHT). The baby remained hemodynamically stable but continued to require CPAP of 7-8 mmHg support in the NICU, despite several unsuccessful attempts to wean her off.

At the age of 3 weeks (30 weeks of corrected gestational age CGA), the echocardiogram revealed a hemodynamically significant PDA measuring 2.2 mm, with nonrestrictive left-to-right flow. The estimated pulmonary artery pressure was approximately 2/3 systemic pressure, with normal biventricular size and function and moderate dilation of left atrium; left ventricle output was high at 378 mL/min/kg. The infant received a 7-day course of acetaminophen to treat the PDA. A follow-up echocardiogram showed a small reduction in the size of the PDA (1.9 mm) and a very mildly restrictive Doppler flow pattern of left-to-right shunt. However, the estimated pulmonary artery pressure was still higher than ⅔ systemic pressure and the right ventricle was moderately dilated. No further treatment was administered to treat the PDA. The medical team discussed that considering the degree of PHT, further medical treatment might not be effective and can be risky especially with the echocardiographic findings of dilated right ventricle.

**Figure 1:**
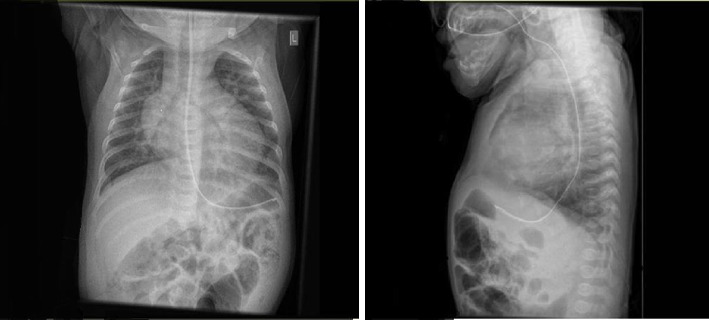
Chest x-ray showed prominence of the cardiothymic silhouette pulmonary vascularity and increased interstitial marking.

At 33 weeks CGA, the infant had an increase in oxygen requirement with a poor gas exchange that needed escalation of respiratory support from CPAP of 8 mmHg to nasal intermittent positive pressure ventilation (NIPPV) with pressures of 18/8 rate of 30/min, and oxygen requirement increased from 28% to 60%. The follow-up echocardiogram showed severe PHT, moderate thickening and dilatation of RV, and moderate PDA of 1.8 mm with mildly restrictive Doppler flow pattern. Despite starting treatment with Aldactazide 3 mg/kg/day for 1 week, PHT worsened. The repeat echocardiogram showed more dilation of RV with a reasonable RV function and moderate PDA; respiratory support was escalated to NIPPV 20/10 to optimize her oxygenation and ventilation. Chest x-ray showed prominence of the cardiothymic silhouette pulmonary vascularity and increased interstitial markings (Figure 1).

During discussions with the cardiology and respirology services, it was concluded that the severity of PHT in this infant could not be solely attributed to the extent of their lung disease. Instead, it was suspected that the pulmonary overcirculation resulting from the PDA shunt might be contributing to this condition. Therefore, the decision was made to proceed with PDA ligation in order to reduce the risk of further cardiopulmonary compromise.

At the age of 35 weeks CGA, the infant had a PDA ligation and lung biopsy. Post-PDA ligation echocardiogram revealed a slight improvement in PHT, with the pulmonary artery pressure estimated to be greater than half systemic pressure and a slight improvement in the RV dilation compared with previous assessments. The infant was extubated on Day 4 post-PDA ligation after she had improvement in respiratory status and was placed on NIPPV with settings of 18/8 and an oxygen requirement of 30% rate of 25/min. However, a repeated Echo conducted a week later still demonstrated high pulmonary pressure.

Genetic service was consulted given the severity of this infant's PHT which was felt to be out of keeping with her lung disease for consideration of a primary etiology of her PHT. Microarray analysis was done and showed Xp22.33 duplication. Specifically, the duplication crosses a gene (ARSL) associated with chondrodysplasia punctata 1, X-linked recessive (CDPX1). This manifests almost exclusively in males and is a genetic disorder present from birth that affects bone and cartilage development. This female infant did not have the phenotypic or radiologic features of this condition. The infant's mother had the same duplication. The genetic team regarded this as inconclusive results. No further genetic testing was done.

**Figure 2:**
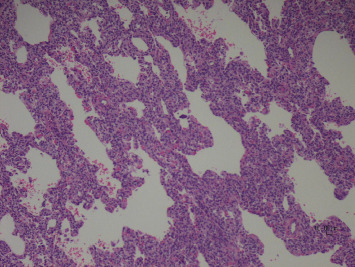
Peripheral wedge biopsy of lung. Diffuse thickening of septa by mesenchymal cells containing glycogen. H&E × 100.

**Figure 3:**
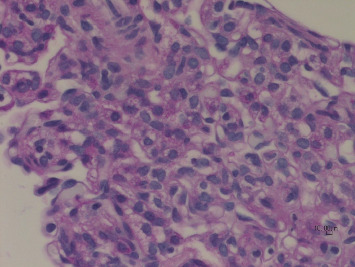
Glycogen-containing mesenchymal cells in the septa. PAS × 600.

**Figure 4:**
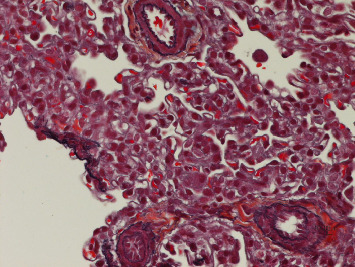
Grade I pulmonary hypertensive changes. Movat × 600.

**Figure 5:**
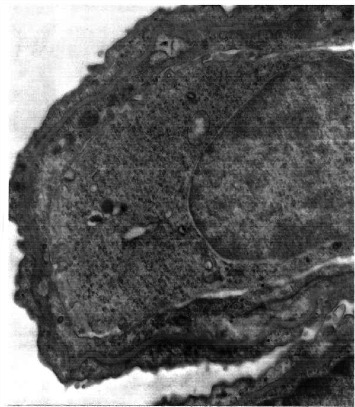
Glycogen granules in the cytoplasm of a pulmonary septal mesenchymal cell. EM × 20,000.

**Figure 6:**
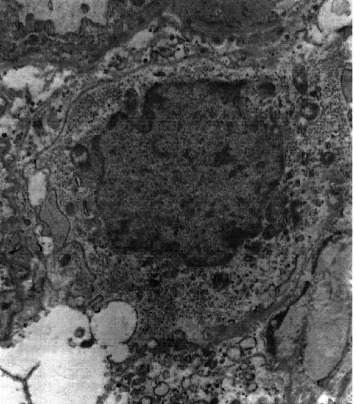
Glycogen granules in the cytoplasm of a pulmonary septal mesenchymal cell. EM × 10,000.

Lung biopsy showed diffuse uniform thickening of the interstitium in the septa between alveoli due to the presence of glycogen-containing macrophages and small mature lymphocytes. The presence of glycogen is verified with the PAS and PASD stains. There was also a population of small mature lymphocytes, very rare fibroblasts, and plasma cells. There were no other inflammatory cells in the interstitial septa or the air spaces. Overall, inflammation did not contribute to the changes seen on microscopy. The microscopic appearance was in keeping with pulmonary interstitial glycogenosis (P.I.G). The lung showed minor, Grade 1 pulmonary hypertensive changes seen as slight hypertrophy of the media of small peripheral pulmonary vessels (Figures 2, 3, 4, 5, and 6).

The infant was treated conservatively; kept on NIPPV of 18/8 mmHg, rate of 20/min and daily Aldactazide; she has subsequently transitioned to CPAP of 8 mmHg at 39 weeks CGA, a week later she was switched to a high-flow nasal cannula 8 L/min that was weaned slowly as the respiratory condition and blood gases improved. She was off all oxygen support at CGA 41 + 5 weeks (103 days of life). Aldactazide was stopped once weaned off respiratory support. A follow-up echocardiogram done prior to discharge showed much improvement with pulmonary artery pressure estimated to be less than 1/2 systemic by TR jet and septal curvature in systole, mild RV hypertrophy, and normal LV size and function. Postdischarge echocardiogram at 8 months and 2 years of age were completely normal.

## 2. Discussion

In this case report, we investigate the presence of severe interstitial lung disease as a cause of respiratory failure in an infant with longstanding PDA. P.I.G is classified as a unique form of neonatal interstitial lung disease [1] that is characterized by evidence of diffuse, uniform interstitial thickening due to the presence of immature interstitial cells with increased levels of cytoplasmic glycogen. Due to the nature of the disease progression, P.I.G is classified in the literature as a developmental abnormality, differentiating it from similar infections and inflammation-based pulmonary disorders [2]. It has variable presentation including tachypnea, hypoxemia, respiratory failure, and PHT. The severity of symptoms can vary from mild to severe, and in some cases, the condition may be discovered incidentally during the evaluation of other respiratory conditions.

The presentation of P.I.G in this case included symptoms of respiratory distress with unsuccessful attempts of removing the patient from respiratory support, and it was an unexpected diagnosis in this preterm infant. Diagnosing P.I.G can pose challenges as it is a rare condition with clinical manifestations that can mimic those of other lung diseases. A lung biopsy is necessary to confirm the diagnosis to identify the distinctive histological features. However, due to the invasive nature of this procedure, lung biopsies are typically reserved for cases where uncertainty remains or after ruling out other pulmonary diseases; this approach may have led to an underestimation of the prevalence of this condition.

The association of P.I.G with congenital heart disease (CHD) and PHT has been described previously; Cutz et al. [3] illustrated the clinical significance of P.I.G when associated with cardiac and pulmonary complications in 28 infants, of which 7 cases died due to increased CHD or respiratory failure complications. In addition, Seidl E. et al. [4] reported and systematically analyzed 11 new cases of P.I.G; eight out of the eleven cases had extrapulmonary comorbidities with congenital heart defects being the most common, and 70% of the patients had PHT. Only two infants were preterm at 30 weeks and 34 weeks of gestational age. The 30-weeks preterm infant and another term infant with mucopolysaccharidosis had PDA. All analyzed lung tissues had clear signs of P.I.G and reduced alveolar growth. Consistent with the published data, the paper highlighted a correlation between P.I.G and extrapulmonary comorbidities or systemic diseases in over 50% of the patients.

The association of P.I.G with a PDA and PHT has also been described previously by Radman et al. [5], mentioning that the clinical case of a neonate with severe PHT during the neonatal period could not only be explained by the diagnosed PDA and CHD of the hypoplastic aortic arch. Jiskoot-Ermers et al. [6] described a similar case of a full-term infant who shortly died after being admitted due to hypoxia and PDA with signs of persistent PHT and no improvement after multiple treatment attempts.

While CHD and PDA do not directly cause P.I.G, they may contribute to its development. They may play a role in its development. Both conditions can lead to PHT, and the resulting hemodynamic changes and hypoxia may worsen abnormal lung development or unmask underlying mesenchymal dysregulation. Additionally, increased mechanical stress on lung tissues caused by volume and pressure overload can activate fibroblast-like cells within the interstitium, contributing to the histologic features characteristic of P.I.G [7]. Some cases of PIG have been reported in infants with CHD, suggesting a possible syndromic or genetic defect [8].

Early recognition of P.I.G in infants with unexplained respiratory distress is crucial to allow for early interventions. However, compared with other pediatric interstitial lung diseases, P.I.G tends to have a favorable prognosis. The main management approach is to provide supportive care including supplemental oxygen and ventilatory support that aim to relieve respiratory distress and improve lung function. In addition, mitigate respiratory infection by optimizing nutrition and supporting growth. The potential benefits of treatment with intravenous or oral corticosteroids may play a role as a treatment option [9, 10]. Their benefit in P.I.G seems to stem more from promoting cellular maturation than from reducing inflammation, making their use highly individualized and depends on the clinical context [1].

In the reported cases, the majority of infants diagnosed with P.I.G received either intravenous or oral corticosteroids, resulting in positive responses. Nevertheless, considering the possible adverse effects of corticosteroids and the limited evidence available from a small number of patients, it is crucial to exercise caution and thoroughly evaluate the need for treatment in each individual case. In our patient's case, although corticosteroid treatment was discussed with the respirology team following biopsy confirmation, it was not reintroduced. The child showed steady clinical improvement after PDA closure, alongside supportive respiratory care and management of PHT. This encouraging trajectory aligns with reports of spontaneous recovery in P.I.G without pharmacologic intervention.

## 3. Conclusion

P.I.G is distinct from most pediatric interstitial lung diseases due to abnormal pulmonary mesenchymal development, resulting in glycogen accumulation within interstitial cells. Typically presenting in newborns with respiratory distress and diffuse interstitial infiltrates, it may also be associated with PHT.

The long-term prognosis for infants with P.I.G is variable; while some infants recover spontaneously within the first year, others may develop persistent respiratory issues and progressive lung disease.

This case broadened the phenotype and added to the current literature new information regarding the importance of considering P.I.G as a potential clinical diagnosis in extremely preterm neonates who present with long-standing PDA and unexplained PHT in the absence of any congenital or genetic causes. Early identification may potentially allow for early diagnosis and, therefore, potential intervention methods such as respiratory management, improved nutrition, and corticosteroids may increase treatment success rates [9].
